# Sex Differences in the Skeletal Muscle Response to a High Fat, High Sucrose Diet in Rats

**DOI:** 10.3390/nu15204438

**Published:** 2023-10-19

**Authors:** Nicholas A. Hulett, Leslie A. Knaub, Sara E. Hull, Gregory B. Pott, Rick Peelor, Benjamin F. Miller, Kartik Shankar, Michael C. Rudolph, Jane E. B. Reusch, Rebecca L. Scalzo

**Affiliations:** 1 Division of Endocrinology, Department of Medicine, University of Colorado School of Medicine (UCSOM), Aurora, CO 80045, USA; nicholas.hulett@cuanschutz.edu (N.A.H.); jane.reusch@cuanschutz.edu (J.E.B.R.); 2 Rocky Mountain Regional Veterans Affairs Medical Center, Aurora, CO 80045, USA; 3 Aging & Metabolism Research Program, Oklahoma Medical Research Foundation, Oklahoma City, OK 73104, USAbenjamin-miller@omrf.org (B.F.M.); 4 Oklahoma City Veterans Affairs Medical Center, Oklahoma City, OK 73104, USA; 5 Department of Pediatrics, Section of Nutrition, University of Colorado School of Medicine (UCSOM), Anschutz Medical Campus, Aurora, CO 80045, USA; kartik.shakar@cuanschutz.edu; 6 Department of Physiology, Harold Hamm Diabetes Center, Oklahoma University Health Sciences Center, Oklahoma City, OK 73104, USA; michael-rudolph@ouhsc.edu; 7 Ludeman Family Center for Women’s Health Research, Department of Medicine, University of Colorado School of Medicine (UCSOM), Aurora, CO 80045, USA

**Keywords:** sex differences, type 2 diabetes, mitochondrial respiration, skeletal muscle, metabolism

## Abstract

Men are diagnosed with type 2 diabetes at lower body mass indexes than women; the role of skeletal muscle in this sex difference is poorly understood. Type 2 diabetes impacts skeletal muscle, particularly in females who demonstrate a lower oxidative capacity compared to males. To address mechanistic differences underlying this sex disparity, we investigated skeletal muscle mitochondrial respiration in female and male rats in response to chronic high-fat, high-sugar (HFHS) diet consumption. Four-week-old Wistar Rats were fed a standard chow or HFHS diet for 14 weeks to identify sex-specific adaptations in mitochondrial respirometry and characteristics, transcriptional patterns, and protein profiles. Fat mass was greater with the HFHS diet in both sexes when controlled for body mass (*p* < 0.0001). Blood glucose and insulin resistance were greater in males (*p* = 0.01) and HFHS-fed rats (*p* < 0.001). HFHS-fed males had higher mitochondrial respiration compared with females (*p* < 0.01 sex/diet interaction). No evidence of a difference by sex or diet was found for mitochondrial synthesis, dynamics, or quality to support the mitochondrial respiration sex/diet interaction. However, transcriptomic analyses indicate sex differences in nutrient handling. Sex-specific differences occurred in PI3K/AKT signaling, PPARα/RXRα, and triacylglycerol degradation. These findings may provide insight into the clinical sex differences in body mass index threshold for diabetes development and tissue-specific progression of insulin resistance.

## 1. Introduction

Type 2 diabetes is a global health crisis, including in the United States where 13% of the population has the disease [1]. Conventional risk factors for developing type 2 diabetes are nutrient overload, obesity, age, sedentary lifestyle, and genetic susceptibility [2]. Mounting evidence points to sex as a key cofactor affecting the development of diabetes [3,4]. For example, at the same BMI, women have lower rates of type 2 diabetes than men [5]. A higher BMI threshold for diabetes development in women is consistent with a model wherein women defend their metabolic health in the context of obesity better than men. Female rodent models are also resistant to diet-induced diabetes, and thus may offer insights into sex differences in carbohydrate metabolism [6]. The reasons for this sex difference are multifactorial and likely the result of the effect of sex on the liver, adipose, and skeletal muscle [7,8,9].

Skeletal muscle health is essential for maintaining glucose homeostasis, accounting for ~80% of postprandial glucose uptake [10,11]. Glucose entering a myofiber may be stored or metabolized through glycolysis and mitochondrial oxidation. Muscle storage capacities are limited, making mitochondrial function essential for glucose homeostasis [10]. Mitochondrial function is partially determined by number and quality. Some studies show altered mitochondrial morphology and respiration during type 2 diabetes while others do not [12,13,14,15,16,17,18]. Sex is an underappreciated variable that may contribute to study differences. Cardiorespiratory fitness, which relies upon skeletal muscle mitochondria, is lower in people with type 2 diabetes. This diabetes-associated impairment in fitness is greater in women than men [19]. We have previously shown that skeletal muscle mitochondrial biogenesis responds in a sex-specific manner to other metabolic stressors such as exercise training [20]. However, little data exist at the molecular and mitochondrial levels to define how skeletal muscle adapts differently between males and females in response to an excess nutrient challenge.

The purpose of this study was to define the sex-specific skeletal muscle mitochondrial adaptations that occur in response to a high fat, high sucrose (HFHS) diet. We hypothesized that skeletal muscle respiration would be lower in females relative to males in response to a HFHS diet, which would be related to mitochondrial biogenesis, dynamics, and transcriptomic differences.

## 2. Materials and Methods

### 2.1. Animals

Four-week-old male and female Wistar rats (Charles River Laboratories International Inc., Wilmington, MA, USA) were randomized to receive standard chow (60% carbohydrate, 17% fat, 23% protein with 5 g sucrose/100 g of diet; Envigo Madison, WI, Custom Diet TD.190369) or a HFHS diet (41% carbohydrate, 39% fat, 20% protein with 45 g sucrose/100 g of diet; Envigo Madison, WI, Custom Diet TD.190370) for 14 weeks. The sources of fat in each diet were lard, coconut oil, and soybean oil. To control for the estrous cycle, female rats were ovariectomized at 12 weeks of age, and estradiol was administered in the drinking water (0.5 μM) from ovariectomy during the project period as previously described [21]. This strategy was employed to limit handling prior to sacrifice for the staging of the female rats. The effectiveness of the estradiol addback was confirmed by comparison to sham ovariectomized females (Supplementary Figure S1). There were no differences in uterine mass (*p* = 0.1913), glucose (*p* = 0.5867), insulin (*p* = 0.78090), and mitochondrial protein synthesis (*p* = 0.5185). The number of animals used for experiments was based on predetermined power analyses and noted below. Body mass (*n* = 18 M-Chow, 18 M-HFHS, 16 F-Chow, 17 F-HFHS), gonadal fat (*n* = 18 M-Chow, 18 M-HFHS, 16 F-Chow, 17 F-HFHS), blood, the liver, and the gastrocnemius were collected at sacrifice. Animals were housed in thermoneutral conditions (30 °C) with a 12:12 light cycle, provided with water and food ad libitum, and fasted 2 h prior to sacrifice [21]. The Institutional Animal Care and Use Committee at the Rocky Mountain Regional VA Medical Center approved all protocols (CD1807R, 13 October 2020).

### 2.2. Blood Collection and Preparation

Animals were anesthetized under 4% isoflurane. Blood was collected via cardiac puncture, spun for 10 min at 3600 rpm and 4 °C, and plasma was collected and saved at −80 °C for glucose (*n* = 10 M-Chow, 8 M-HFHS, 10 F-Chow, 11 F-HFHS) (Cayman Chemicals, item #10009582, Ann Arbor, MI, USA) and insulin (*n* = 9 M-Chow, 8 M-HFHS, 9 F-Chow, 11 F-HFHS) (ALPCO Diagnostics, Catalogue #80-INSRT-E01, Salem, NH, USA) assays. The homeostasis model assessment for insulin resistance (HOMA-IR) (*n* = 9 M-Chow, 8 M-HFHS, 10 F-Chow, 10 F-HFHS) was calculated using previously described methods [22].

### 2.3. Mitochondrial Respiration

Mitochondrial respiration was measured using Oroboros Oxygraph-2k (O2k, Oroboros Instruments Corp., Innsbruck, Austria) according to modifications from previously described protocols [23,24,25,26]. Immediately after removal, the gastrocnemius was placed in ice-cold mitochondrial preservation buffer (BIOPS (10 mM Ca-EGTA, 0.1 mM free calcium, 20 mM imidazole, 20 mM taurine, 50 mM K-MES, 0.5 mM DTT, 6.56 mM MgCl_2_, 5.77 mM ATP, 15 mM phosphocreatine, pH 7.1)). Muscle fibers were separated mechanically (in BIOPS and on ice), then permeabilized by incubation with saponin (40 μg mL^−1^) in BIOPS on ice on a shaker (30 min), then washed in mitochondrial respiration buffer (MiR06 (0.5 mM EGTA, 3.0 mM magnesium chloride, 60 mM K-lactobionate, 20 mM taurine, 10 mM potassium phosphate, 20 mM Hepes, 110 mM sucrose, 1 g L^−1^ bovine serum albumin, 280 U mL^−1^ catalase, pH 7.1)). Next, 1.5–2 mg of fibers were added to pre-warmed MiR06 + 25 μM blebbistatin in the O_2_k. Oxygen in the MiR06 was started at 400 μM and maintained at >250 μM.

Two sets of substrates and inhibitors were added to assess respiration rates at several states in duplicate. Rates for Run 1 were measured following the addition of pyruvate (P, 5 mm) and malate (M, 1 mM) (LEAK_PM_); PM with ADP (2 mM) (OXPHOS_PM_); PM, ADP, glutamate (G, 10 mM PMG P), and succinate (S, 10 mM) (OXPHOS_PMGS_); PMGS, ADP, and oligomycin (2 μg mL^−^^1^, LEAK_PMGS_). Rates for Run 2 were measured following the addition of Palmitoylcarnitine (PC, 50 μM) and malate (M, 1 mM) (LEAK_PCM_); PCM with ADP (2 mM) (OXPHOS_PCM_); PCM, ADP, and succinate (S, 10 mM) (OXPHOS_PCMS_); PCMS, ADP, and oligomycin (2 μg mL^−1^) (LEAK_PCMS_). Both runs ended with 0.5 μM stepwise titration of 4-(trifluoromethoxy) phenylhydrazone (FCCP) until maximal uncoupling was achieved (electron transfer system capacity (E)). Cytochrome c (10 μM) was also added during both runs to determine that mitochondrial membrane damage was minimal and the same between runs. Runs were accepted with a less than 5% increase with cytochrome c addition. Respiration ratios were calculated as a ratio of LEAK/OXPHOS_PM_, LEAK/OXPHOS_PCM_, LEAK/OXPHOS_PMGS_, LEAK/OXPHOS_PCMS_, OXPHOS/Uncoupled_PMGS_, and OXPHOS/Uncoupled_PCMS_ (*n* = 10 M-Chow, 10 M-HFHS, 10 F-Chow, 12 F-HFHS for all respiration measurements).

### 2.4. Western Blotting

The gastrocnemius was flash frozen for the determination of protein content of targets of interest. Tissues were powdered with liquid nitrogen in lysis buffer (MPER (Thermo Scientific, Waltham, MA, USA) with 150 mmol/L NaCl, 1 mmol/L EDTA, 1 mmol/L EGTA, 5 mmol/L Na_4_P_2_O_7_ 10H_2_O), 1 mmol/L Na_3_PO_4_, 20 mmol/L NaF, 500 mmol/L okadaic acid, 1% protease inhibitor cocktail (Sigma Aldrich, St. Louis, MO, USA)), and further processed with a rotor-stator homgenizer. Lysate protein concentration was determined by the Bradford method and equal protein was resolved on 4–15% SDS-PAGE gels followed by transfer to a PVDF membrane. Data for each target were collected across multiple blots due to the number of samples and the constraints of gel capacities. Each blot included samples from each group to avoid blot-to-blot bias. Equal protein loading and transfer were confirmed with ponceau staining. Cross-blot variability was controlled with a consistent loading control sample with which data were normalized. Membranes were probed with specific antibodies for peroxisome proliferator-activated receptor gamma coactivator 1-alpha (PGC1α, RRID:AB_881987, 1:500) (*n* = 9 M-Chow, 9 M-HFHS, 10 F-Chow, 12 F-HFHS), total endothelial nitric oxide synthase (eNOS, RRID:AB_329863, 1:500) (*n* = 9 M-Chow, 10 M-HFHS, 5 F-Chow, 6 F-HFHS), Ser1177 phosphorylated eNOS (peNOS, RRID:AB_329837, 1:500) (*n* = 9 M-Chow, 10 M-HFHS, 5 F-Chow, 6 F-HFHS), total 50 AMP activated protein kinase (AMPK, RRID:AB_330331, 1:500) (*n* = 9 M-Chow, 10 M-HFHS, 10 F-Chow, 11 F-HFHS), Thr172 phosphorylated AMPK (pAMPK, RRID:AB_330330, 1:500) (*n* = 9 M-Chow, 10 M-HFHS, 10 F-Chow, 11 F-HFHS), mitofusin-1 (Mfn1, RRID:AB_11142211, 1:500) (*n* = 10 M-Chow, 11 M-HFHS, 10 F-Chow, 10 F-HFHS), mitofusin-2 (Mfn2, RRID:AB_10999860, 1:500) (*n* = 10 M-Chow, 11 M-HFHS, 11 F-Chow, 10 F-HFHS), mitochondrial fission factor (MFF, RRID:AB_2734126, 1:500) (*n* = 10 M-Chow, 11 M-HFHS, 11 F-Chow, 10 F-HFHS), dyamin-related protein 1 (Drp1, RRID:AB_11178663, 1:500) (*n* = 10 M-Chow, 11 M-HFHS, 11 F-Chow, 10 F-HFHS), phosphorylated Drp1 (pDRP1, RRID:AB_2085352, 1:500) (*n* = 10 M-Chow, 11 M-HFHS, 11 F-Chow, 10 F-HFHS), NAD-dependent siturin-1 (SIRT-1, RRID:AB_2617130, 1:500) (*n* = 10 M-Chow, 11 M-HFHS, 11 F-Chow, 10 F-HFHS), manganese superoxide dismutase (MnSOD, RRID:AB_300434, 1:500) (*n* = 8 M-Chow, 9 M-HFHS, 8 F-Chow, 7 F-HFHS), Sestrin2 (Ses2, RRID:AB_11178663, 1:500) (*n* = 10 M-Chow, 10 M-HFHS, 11 F-Chow, 10 F-HFHS), Bcl-2 interacting protein (BNIP3, RRID:AB_2259284, 1:500) (*n* = 7 M-Chow, 8 M-HFHS, 9 F-Chow, 8 F-HFHS), PTEN induced putative kinase 1 (PINK1, RRID:AB_11179069, 1:500) (*n* = 10 M-Chow, 11 M-HFHS, 10 F-Chow, 10 F-HFHS), Total OXPHOS Rodent WB Antibody Cocktail (RRID:AB_2629281, 1:500) (*n* = 9 M-Chow, 10 M-HFHS, 10 F-Chow, 12 F-HFHS), and α-tubulin (RRID:AB_2241126, 1:1000) (*n* = 10 M-Chow, 11 M-HFHS, 10 F-Chow, 10 F-HFHS). Proteins were detected by fluorescent secondary antibodies and detected/visualized by Odyssey CLX (LICOR Biotechnologies, Lincoln, NE, USA). Western blot scans and densitometric analysis were performed using Image Studio v4.1.

### 2.5. RNA Sequencing

Rat gastrocnemius tissue (50–100 mg) was homogenized in 1 mL of Trizol (Invitrogen, Waltham, MA, USA) using the Bead Mill 24 using a speed of 5.50 m/s, cycles of 2, a cycle time of 0:30 sec, and a dwell between run times of 0:30 s. Total RNA was isolated using a combination of TRI reagent (Molecular Research Center, Cincinnati, OH, USA) and RNeasy-mini columns, including on-column deoxyribonuclease digestion (Qiagen, Germantown, MD, USA). Directional cDNA libraries for mRNA-sequencing were prepared using polyA-mRNA from individual RNA samples using Illumina TruSeq reagents [27]. Briefly, 1 μg of total RNA was utilized for mRNA isolation using poly-A magnetic beads and was fragmented prior to first- and second-strand cDNA synthesis. Libraries were generated via PCR including dual-indexed barcodes. Following purification with AmpureXP beads, libraries were quantitated using Qubit dsDNA reagents (Invitrogen, Waltham, MA, USA). Pooled cDNA libraries were sequenced (150 bp paired reads) using a NovaSeq 6000 instrument (Illumina, San Diego, CA, USA) by the University of Colorado Cancer Center Genomics and Microarray core facility. All raw sequencing files may be obtained using GEO id: GSE228215 (https://www.ncbi.nlm.nih.gov/geo/, accessed on 7 September 2023).

Following sequencing and demultiplexing, reads were trimmed for adapters and filtered based on quality score using Fastp as previously described [28]. High-quality reads were aligned to the rat genome (rn6) using STAR (ver 2.7.6a) using the default settings. The resulting read alignments file for each sample was imported in Seqmonk (ver 1.48.1) for transcript-level quantification as counts mapping to annotated genes. The raw counts matrix of all genes was imported into DESeq2 (DESeq2 version 1.30.1) in R (ver 4.1), which was used to normalize, Log2 transform, and calculate significant differentially expressed genes (DEG). Quantitation was from the Seqmonk RNA-Seq pipeline on merged transcripts, counting reads over exons as raw counts. All genes with count values greater than 0.1 were used in DESeq2 to calculate significant DEG (*p* < 0.05) within females or within males by dietary group. Normalized, log2 transformed significant DEGs were then seeded into Ingenuity Pathway Analysis (Qiagen Inc., www.qiagenbioinformatics.com) to calculate significantly enriched pathways within females or males by dietary group that are in common between groups.

### 2.6. Tissue Preparation and Determination of Mitochondrial Fractional Synthesis Rate

A subset of animals was labeled for the measurement of mitochondrial biogenesis (*n* = 6 M-Chow, 6 M-HFHS, 6 F-Chow, 5 F-HFHS). The fractional synthesis rate of mitochondrial proteins was performed and described previously [20]. Rats received a single intraperitoneal injection of 99% enriched D_2_O (Sigma-Aldrich, St. Louis, MO) calculated to enrich the body water pool (assumed 60% of body weight) to 5% and given ad libitum access to drinking water enriched to 8% one week before sacrifice [29]. Flash-frozen gastrocnemius was homogenized, fractionated by differential centrifugation, hydrolyzed, and derivatized by our previously published methods [20]. Derivatized amino acids were analyzed on Agilent 7890B GC and 5977B mass spectrometers. Body water deuterium enrichment was determined in plasma using a liquid water isotope analyzer (Los Gatos Research, Los Gatos, CA, USA) against a standard curve prepared with samples containing different concentrations of D_2_O. Mitochondrial protein fractional synthesis rate (FSR, %/day) was calculated using the enrichment of alanine in mitochondria divided by the precursor enrichment (as determined by the body water pool with adjustment for equilibrium with alanine) divided by time.

### 2.7. Statistical Analysis

Data were analyzed with two-way analysis of variance (ANOVA); diet (chow versus HFHS) and sex (male versus female) were the two factors. Pairwise comparisons were performed using the Tukey test where appropriate. Multiple linear regression was used to determine the impact of sex and diet on gonadal fat mass and liver with body mass as an additional factor. For RNA sequencing, all genes with count values >0.1 were used to calculate significant DEG for the interaction between sex and diet, and then within females or within males by dietary group (*p* < 0.05 FDR, Benjamimi and Hochberg). The level of statistical significance was set at *p* < 0.05 for all data. Data are expressed as mean ± SE. All calculations were performed using GraphPad Prism (version 9.4.0 for Windows, GraphPad Software, San Diego, CA, USA). The datasets generated during and/or analyzed during the current study are available from the corresponding author upon reasonable request.

## 3. Results

### 3.1. Animal Characteristics

**Figure 1:**
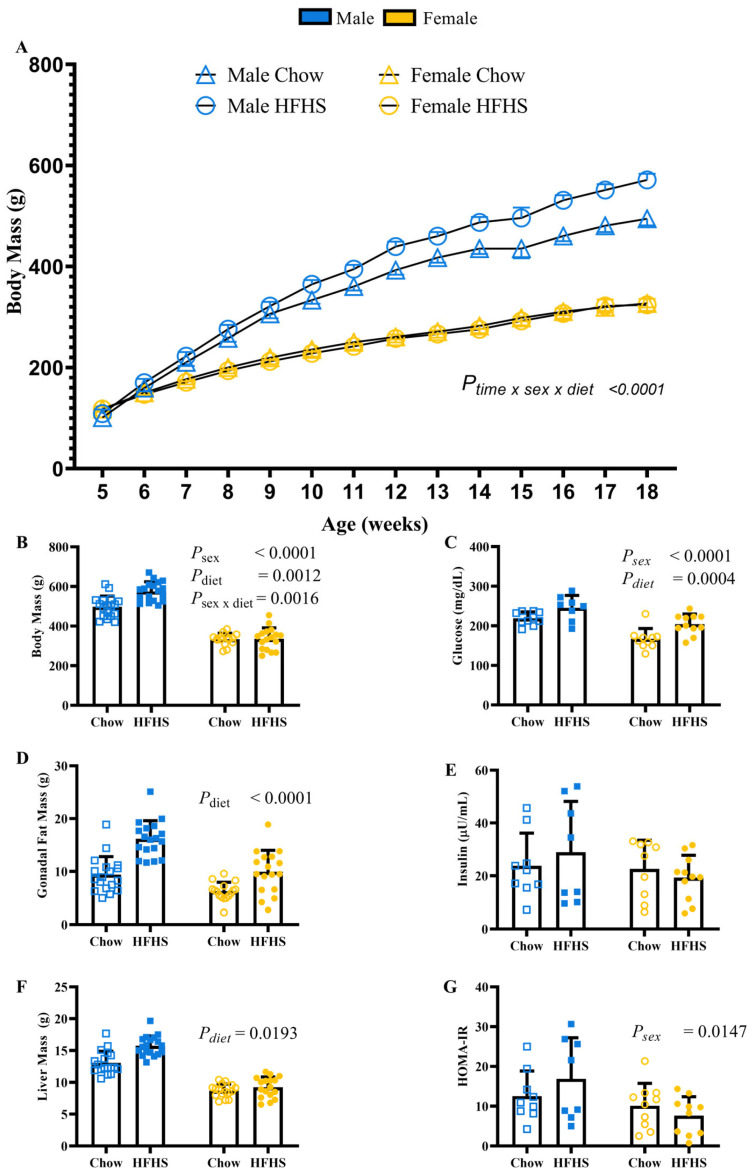
Animal Characteristics. (**A**), Body Mass from the beginning of dietary intervention through 14 weeks (mean ± SD). Blue open squares = Males on chow diet, blue closed squares = Males on HFHS diet, yellow open circles = Females on chow diet, yellow closed circles = females on HFHS diet. (**B**), Body mass 14 weeks post dietary intervention (*n* = 18, 18, 16, 17, respectively, for M Chow, M HFHS, F Chow, and F HFHS) (**C**), Blood Glucose 14 weeks post dietary intervention (*n* = 10, 8, 10, 12, respectively) (**D**), Gonadal Fat mass 14 weeks post dietary intervention (*n* = 18, 18, 16, 17, respectively) (**E**), Blood insulin 14 weeks post dietary intervention (*n* = 9, 8, 9, 11, respectively) (**F**), Liver mass 14 weeks post dietary intervention (*n* = 18, 18, 16, 17, respectively) (**G**), Homeostatic Model Assessment for Insulin Resistance (HOMA-IR) 14 weeks post dietary intervention (*n* = 9, 8, 10, 10, respectively). In panels A and C-G, blue boxes represent males and yellow circles represent females. Open shapes represent chow fed animals and closed shapes represent HFHS fed animals. Chow = standard diet, HFHS = high-fat high-sugar diet.

At sacrifice, males fed the HFHS diet gained more body weight over time compared with females on the HFHS diet (Figure 1A; *p* < 0.0001 for the interaction of time, diet, and sex). Males on the HFHS diet weighed more relative to chow-fed males and to females on either diet (Figure 1B; *p* = 0.0010 for the sex by diet interaction). Both gonadal fat mass and liver mass were greater with the HFHS diet when body mass was controlled for statistically (Figure 1C,D; *p* < 0.0001 and *p* = 0.0193, respectively). Sex did not influence these outcomes (*p* > 0.2973). Blood glucose was affected by both sex (Figure 1E; *p* < 0.0001), where males had higher levels than females, and diet (*p* = 0.0004), where both males and females on the HFHS diet had higher levels than chow-fed animals. Insulin resistance, estimated by HOMA-IR, was higher in males (Figure 1G; *p* = 0.0147) but unaffected by diet (*p* = 0.1414). No difference in insulin concentration was observed by sex (Figure 1F; *p* = 0.8333) or diet (*p* = 0.2162).

### 3.2. Skeletal Muscle Mitochondrial Respiration

**Figure 2:**
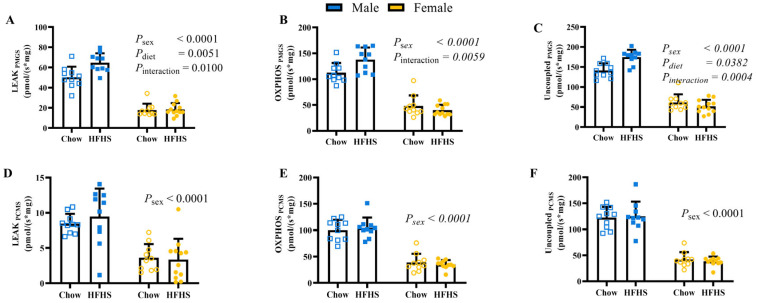
Mitochondrial Respiration measured by Oroboros O2k. (**A**,**D**), O_2_ consumption during leak state with carbohydrate-linked or lipid-link substrate addition, respectively. (**B**,**E**), O_2_ consumption during OXPHOS state with carbohydrate-linked or lipid-link substrate addition, respectively. (**C**,**F**) O_2_ consumption during Uncoupled state with carbohydrate-linked or lipid-link substrate addition, respectively. (P: pyruvate; M: malate; G: glutamate; S: succinate (**A**,**C**,**E**); (**C**) octanolycarnitine; M: malate; G: glutamate; S: succinate (**B**,**D**,**F**)). Chow = standard diet, HFHS = high-fat high-sugar diet. *n* = 10 for M Chow, M HFHS, and F Chow, *n* = 12 for F HFHS. Blue boxes represent males and yellow circles represent females; Open shapes represent chow fed animals and closed shapes represent HFHS fed animals. Chow = standard diet, HFHS = high-fat high-sugar diet.

Leak (L), OXPHOS (P), and uncoupled (E) respiration using carbohydrate-linked substrates were greater in males in the context of the HFHS diet but unaffected by diet in females (Figure 2A–C; *p* < 0.0001 for sex by diet interaction). Significant sex differences observed for lipid-linked substrate respiration was enhanced in L, P, and E in males relative to females (Figure 2D–F; *p* < 0.0001). However, the sex by diet interaction significance was lost when using lipid-linked substrates for mitochondrial respiration. L and P states as well as ratios between states can be found in Table 1.

### 3.3. Skeletal Muscle Mitochondrial Biogenesis and Protein Content

**Figure 3:**
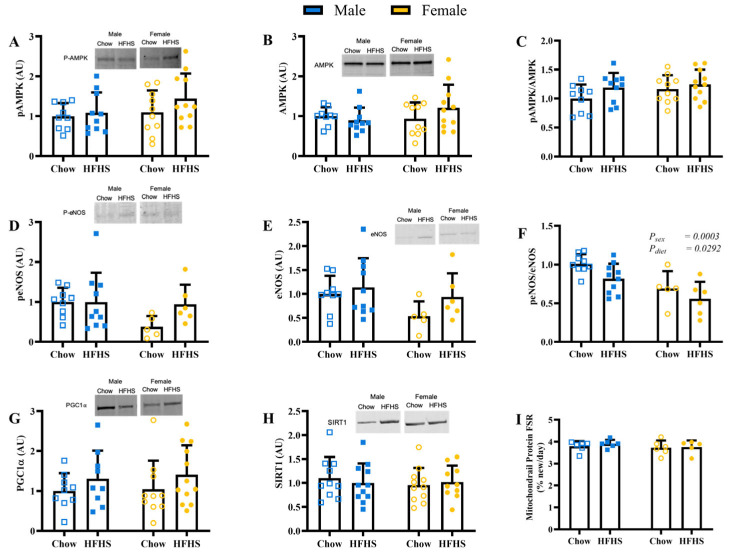
Skeletal Muscle Mitochondrial Biogenesis. (**A**), protein content of phosphorylated AMP-activated protein kinase (pAMPK) (*n* = 9, 10, 10, 12, respectively, for M Chow, M HFHS, F Chow, and F HFHS). (**B**), protein content of AMP-activated protein kinase (AMPK) (*n* = 9, 10, 10, 12, respectively). (**C**), ratio of pAMPK to AMPK (*n* = 9, 10, 10, 12, respectively). (**D**), protein content of phosphorylated endothelial nitric oxide synthase (peNOS) (*n* = 9, 10, 5, 6, respectively). (**E**), protein content of endothelial nitric oxide synthase (eNOS) (*n* = 9, 10, 5, 6, respectively). (**F**), ratio of peNOS to eNOS (*n* = 9, 10, 5, 6, respectively). (**G**), protein content of peroxisome proliferator-activated receptor gamma coactivator-1 alpha (PGC1α) (*n* = 9, 9, 10, 12, respectively). (**H**), protein content of sirtuin 1 (SIRT1) (*n* = 10, 11, 11, 10, respectively). (**I**), Mitochondrial protein fractional synthesis rate (FSR) determined by deuterium labeling (*n* = 6, 6, 6, 5, respectively). Blue boxes represent males and yellow circles represent females; Open shapes represent chow fed animals and closed shapes represent HFHS fed animals. Chow = standard diet, HFHS = high-fat high-sugar diet.

There was no difference between sexes or diets in total, phosphorylation, or the ratio of phosphorylated to total AMPK (Figure 3A–C; *p* > 0.2511), a key cellular energy sensor. There were also no differences in phosphorylated or total endothelial nitric oxide (eNOS) (Figure 3D,E, *p* > 0.0842). However, effects of sex and diet were detected in the ratio of phosphorylated endothelial nitric oxide to total (Figure 3F; *p* = 0.0003 & *p* = 0.0292, respectively). There were also no differences between groups in protein levels of PGC1α or SIRT1 (Figure 3G,H; *p* > 0.4991). Furthermore, we did not detect any differences in mitochondrial protein fractional synthesis rate between groups (Figure 3I; *p* > 0.3412).

### 3.4. Skeletal Muscle Mitochondrial Quality

**Figure 4:**
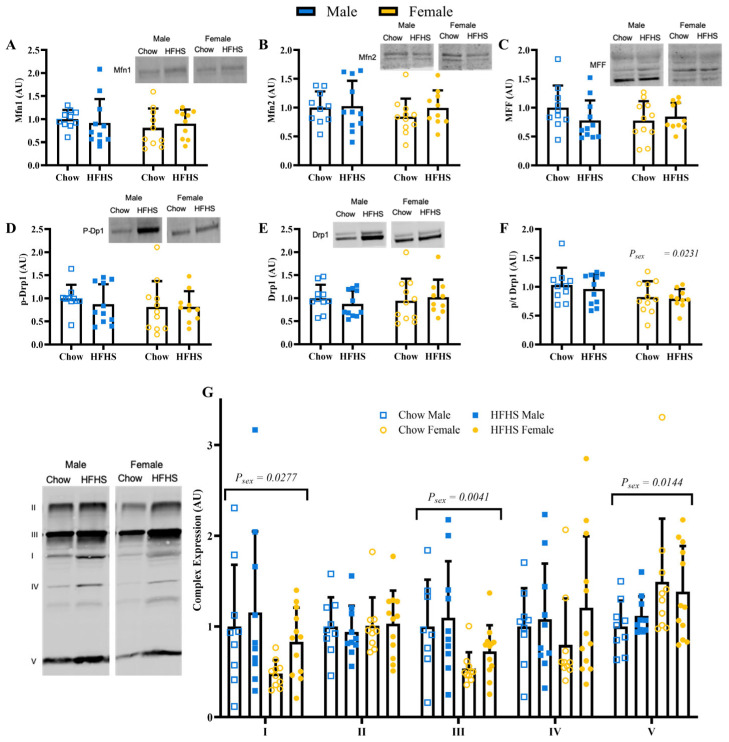
Skeletal Muscle Mitochondrial Dynamics. (**A**), Protein content of Mitofusin-1 (Mfn1) (*n* = 10, 11, 10, 10, respectively, for M Chow, M HFHS, F Chow, and F HFHS). (**B**), Protein content of mitofusin-2 (Mfn2) (*n* = 10, 11, 11, 10, respectively). (**C**), Protein content of mitochondrial fission factor (MFF) (*n* = 10, 11, 11, 10, respectively). (**D**), protein content of phosphorylated dynamin-related protein 1 (pDrp1) (*n* = 10, 11, 11, 10, respectively). (**E**), protein content of dynamin-related protein 1 (Drp1) (*n* = 10, 11, 11, 10, respectively). (**F**), ratio of pDrp1 to total Drp1 (*n* = 10, 11, 11, 10, respectively). (**G**), protein content of mitochondrial complexes (*n* = 9, 10, 10, 12, respectively). Blue boxes represent males and yellow circles represent females; Open shapes represent chow fed animals and closed shapes represent HFHS fed animals. Chow = standard diet, HFHS = high-fat high-sugar diet.

**Figure 5:**
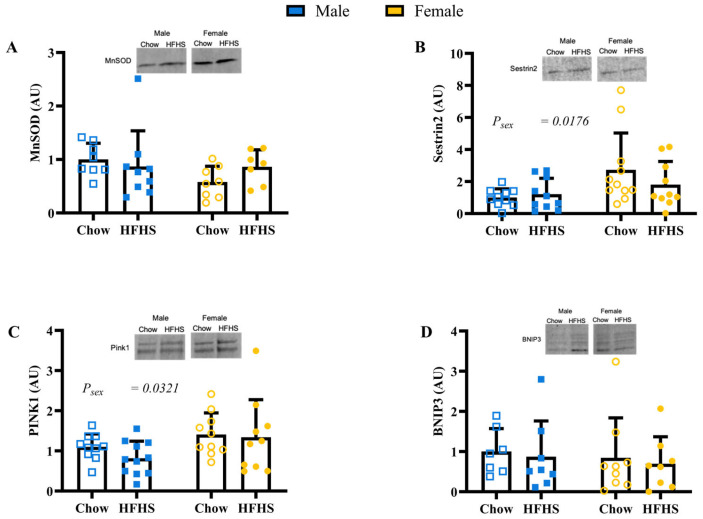
Skeletal Muscle Mitochondrial Health. (**A**), protein content of manganese superoxide dismutase (MnSOD) (*n* = 8, 9, 8, 7, respectively, for M Chow, M HFHS, F Chow, and F HFHS). (**B**), protein content of Sestrin2 (*n* = 10, 10, 11, 10, respectively). (**C**), protein content of PTEN-induced kinase 1 (PINK1) (*n* = 10, 11, 10, 10, respectively). (**D**), protein content of Bcl-2-interacting protein 3 (BNIP3) (*n* = 7,8,9,8, respectively). Blue boxes represent males and yellow circles represent females; Open shapes represent chow fed animals and closed shapes represent HFHS fed animals. Chow = standard diet, HFHS = high-fat high-sugar diet.

Expression of Mfn1, Mfn2, MFF, phosphorylated DRP1, and total DRP1 were not different by sex or diet (Figure 4A–E; *p* > 0.1677). The ratio of phosphorylated to total DRP1 was greater in males (Figure 4F; *p* = 0.0231). Males had greater expression of complexes I and III (Figure 4G; *p* = 0.0277 and *p* = 0.0041, respectively). Females had greater expression of complex V (Figure 4G; *p* = 0.0144). Sex and diet did not have an effect on complexes II and IV. MnSOD and BNIP3 were not different between groups (Figure 5A,D; *p* > 0.1823). Sestrin2 and PINK1 protein content were higher in females (Figure 5B,C; *p* = 0.0176 and *p* = 0.0321, respectively).

### 3.5. RNA Sequencing

**Figure 6:**
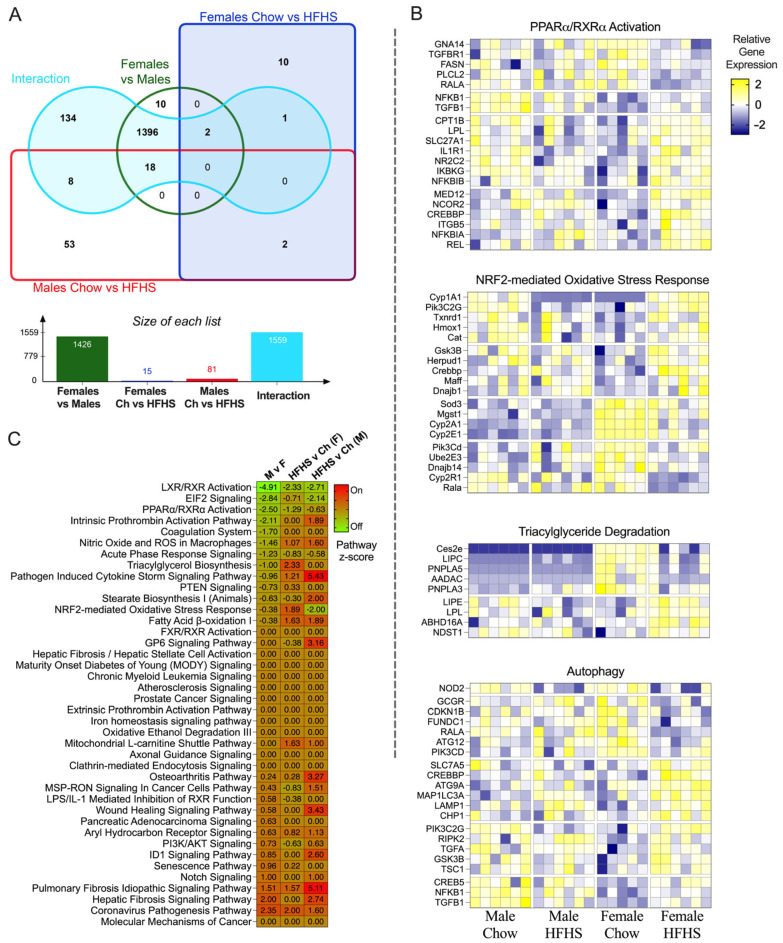
RNA Sequencing. (**A**), Venn Diagram and quantification of the number of transcripts shared between group pairs. (**B**), Significance of pathway enrichment by group. (**C**), Relative expression of genes within the PI3K/AkT Signaling Pathway, NRF2- Mediated Oxidative Stress Pathway, Triacylglycerol Degradation Pathway, and Autophagy Pathway. Chow = standard diet, HFHS = high-fat high-sugar diet.

Differences between sex and dietary response were evaluated by bulk RNA sequencing of skeletal muscle, as an exploratory experiment, to decipher potential mechanisms of the sex differences in mitochondrial respiration and to generate future hypotheses. There were overall transcriptomic differences by sex. In total, 1426 genes were differentially expressed between males and females. There was also an interaction of diet and sex in the transcript data with 1559 genes differentially regulated by diet depending on the sex (Figure 6A). Ingenuity Pathway Analysis was used to identify pathways related to these genes. Here, we revealed significant diet and sex differences in LXR/RXR Activation, EIF2 Signaling, PPARα/RXRα Activation, the Fibrosis Idiopathic Signaling Pathway, Notch Signaling, and the Senescence Pathway (Figure 6B). To further investigate sex differences in response to a HFHS diet, we examined the genes within the regulatory pathways of interest, including PPARα/RXRα Activation, NRF2-mediated Oxidative Stress Response, Triacylglyceride Degradation, and Autophagy, to identify potential targets for future studies (Figure 6C). Together, these data suggest that in response to the HFHS diet, females tended to have greater expression of genes related to nutrient sensing/storage than males while males have greater expression of genes related to inflammation.

## 4. Discussion

Here, we report sex-specific metabolic differences in rats challenged with a HFHS diet. Males on a HFHS diet had greater body weight and insulin resistance while females did not differ from chow-fed rats. Gonadal fat was greater with a HFHS diet regardless of sex. A primary finding of this study is an interaction of sex and diet on carbohydrate-linked mitochondrial respiration, where males responded to the HFHS diet with greater respiration while females did not. To uncover the mechanism for mitochondrial respiration and the interaction with sex and diet, we assessed several key mitochondrial parameters. After 14 weeks of the HFHS diet, we observed no evidence of differences in measures of protein markers of autophagy, mitochondrial biogenesis, or mitochondrial dynamics. We did note differences in subunits of the complexes of the electron transport chain. Exploratory RNA sequence analysis revealed that females had greater expression of nutrient storage pathways while males had greater activation of pathways related to inflammation with a HFHS diet. Together, these data demonstrate sex differences in response to a HFHS diet, where females have lower carbohydrate-linked mitochondrial respiration that is not explained by mitochondrial content or dynamics.

High-fat feeding augments mitochondrial biogenesis [29]. This process is initiated by cellular nutrient sensing and activation of PGC-1α which can be considered the “master regulator of mitochondrial biogenesis” [30]. Sex differences in PGC-1α expression have been observed. For example, women have greater PGC-1α transcript expression after a bout of exercise [31]. In addition, PGC-1α and mtDNA content were higher in both male and female Wistar rats after a 26-week high-fat diet [32]. In the current study, however, there was no evidence of a difference in PGC-1α expression or content by sex or diet. This interstudy difference may be due to housing temperature differences between studies (22 °C room temperature versus 30 °C rat thermoneutrality), which has been shown to alter metabolic phenotypes [33]. Recent work highlights the complexity of mitochondrial biogenesis, suggesting vital roles for signaling networks beyond PGC-1α [34]. These pathways rely upon nuclear factor erythroid 2-related factor 2 (Nrf2) and estrogen-related receptor gamma (ERRγ) [35]. We did observe differential expression of transcripts within the Nrf2-mediated oxidative stress response pathway. However, there was no difference in the fractional synthesis rate of mitochondrial proteins as measured during the last week of the experiment. The role of estrogen in our model remains open and will be investigated in future studies. We observed sex differences in the content of mitochondrial complexes that were not different between diets. Males had greater complexes I and III content while females had greater content of complex V. Complexes I and III are key sites for carbohydrate metabolism and may explain the sex difference in carbohydrate-linked respiration seen in this study. The difference in content could represent differences in mitochondrial biogenesis which occurred prior to deuterium labeling or accumulation of low-quality mitochondria due to low mitophagy. The autophagy pathway was downregulated in males compared with females in the RNA sequence analysis. Together, these data suggest that mitochondrial biogenesis in the last 7 days of the diet exposure did not contribute to the sex difference in respiration with the HFHS diet.

Mitochondrial dynamics such as fission and fusion are key for mitochondrial health in skeletal muscle and are shaped by sex [36,37]. Mitochondrial dynamics are coordinated through changes in the expression of MFN1, MFN2, and MFF or post-transcriptional modifications in the case of DRP1 [12,38]. Deleting these proteins results in mitochondrial dysfunction, accumulation of mitochondrial DNA damage, stunted growth, increased oxidative stress, and atrophy [39,40,41]. Energetic stressors such as dietary manipulations or exercise training can induce mitochondrial dynamic changes. DRP1 expression was greater with a high-fat diet while there was no difference in MFN2 levels in Sprague-Dawley rats [42]. Sex differences in mitochondrial dynamics have been reported after eccentric resistance training. MFN1, DRP1, and PINK1 were greater in men than in women, authors attributed these differences to sex hormones [43]. In response to the HFHS diet, we observed no evidence of differences in these key elements of mitochondrial dynamics or the upstream signaling molecule sirtuin1 at 14 weeks. Mitochondrial morphology has been shown to be altered with hyperglycemia [44]. As the male rats in our study had higher blood glucose levels, we were surprised at the lack of differences in proteins related to mitochondrial morphology between groups. Fission and fusion are both dynamic events that may not be well captured by single time point evaluations. It is feasible that adaptive changes caused by a HFHS diet occur before 14 weeks and persist, resulting in a new steady state.

We are not the first to report differences in mitochondrial respiration and abundance with respect to sex. The skeletal muscle mitochondrial protein content of Complexes I and V was greater in female rats at baseline, with no change in response to caloric restriction [45]. Studies report higher levels of mitochondrial content and respiration in females across multiple tissues including skeletal muscle and liver [8,46,47,48]. The current study agrees with greater expression of Complex V in females; however, this difference in complex content did not result in greater respiration. While the mechanism underpinning this difference remains unclear, two factors that influence respiration are substrate delivery and signal sensitivity. Tracer studies show that lipid uptake into skeletal muscle is higher in females compared to males [49]. A major fuel source of mitochondria is triglycerides, which must be broken down by lipoprotein lipase (LPL) to fatty acids and glycerol before it can enter the cell and oxidation can occur. We measured greater LPL transcript abundance in females compared to males in agreement with the human literature [50]. Future studies should examine if this transcript change reflects protein LPL levels as well as the activation state of LPL. In contrast, others report that females have moderately lower mitochondrial ADP sensitivity with no difference in substrate transport [51]. Our study design does not allow us to comment on ADP sensitivity as our measurements were taken at a constant ADP concentration (2mM). We plan to test for mitochondrial sensitivity by using multiple ADP concentrations in future studies.

Our respiration data suggest that males respond to a HFHS diet with greater maximal rates of nutrient oxidation in skeletal muscle while females do not. Nutrient excess can set the stage for a mismatch between supply and demand within the mitochondria. For example, oxidizing an incoming substrate without adequate demand creates a high protonmotive force which can lead to the generation of reactive oxygen species and inflammation [52]. These factors are suggested as the primary drivers of insulin resistance in skeletal muscle and may contribute to the difference in insulin resistance in male rats observed in this study [52]. In contrast, the female-specific response of unaltered mitochondrial respiration with a HFHS diet could prevent oxidative damage and subsequent insulin resistance. Additionally, greater content of Sestrin2 and Pink1 as well as greater autophagy transcript expression could align with higher mitophagy in females whereas males adapt with energy production at the cost of greater superoxide production and reduced mitochondrial health. Future studies are needed to evaluate the functional implications for the transcript and protein data.

Several aspects of the study design warrant further discussion. Skeletal muscle contains various fiber types with known differences in mitochondrial content. An underlying sex difference in fiber type abundance could explain mitochondrial respiration differences. In humans, females tend to have more oxidative fibers than males [47]. However, this is not the case with rats. While fiber type was not measured in the current study, adult Wistar rats have been shown to have ~1% difference in fiber type between sexes [53]. Another difference between humans and rats is growth characteristics. Rats, especially males, continue to gain weight throughout their lifetime [54]. Increases in lean mass require mitochondrial biogenesis which may overshadow changes caused by the HFHS diet. However, our group has observed interventional mitochondrial respiration differences using this rat model previously [24]. In the current study, we gathered data 14 weeks after diet initiation. This study design was chosen to ensure the animals had adapted to the diet. Compensation likely occurs early in the diet exposure and may have reached a new equilibrium by 14 weeks. The observation of similar mitochondrial biogenesis between sexes suggests that skeletal muscle adaptation occurred prior to measurement in this study. It is also of note that these measurements represent the resting state. One could speculate that under sedentary cage-dwelling conditions, higher skeletal muscle oxidative flux is a maladaptive response. Future studies should define the time course of skeletal muscle adaptation as well as uncover the signaling mechanisms for the changes seen at 14 weeks. Future studies will examine the role of sex hormones, specifically estrogen, on mitochondrial responses to a HFHS diet.

## 5. Conclusions

In conclusion, skeletal muscle mitochondrial respiration adapts to a HFHS diet in a sexually dimorphic manner. A HFHS diet increased weight and insulin resistance in male rats but not female rats. Males have greater carbohydrate-linked respiration while female respiration is diminished. This difference does not appear to be linked to mitochondrial biogenesis, fission, fusion, or quality in the skeletal muscle. As such, these findings suggest differences in systemic fuel handling. A full understanding of sex differences in the systemic fuel partitioning response to a HFHS diet would allow for sex-specific approaches to optimize systemic and skeletal muscle health and metabolic flexibility.

## Figures and Tables

**Table 1:** Mitochondrial Respiration continued. All values are expressed as mean ± standard deviation with a unit of pmol/(s*mg). s, d, i represent statistical significance of sex, diet, and interaction of sex and diet, respectively. L: Leak, P: OXPHOS, E: Uncoupled, P: pyruvate, M: malate, C: octanolycarnitine G: glutamate, S: succinate.

	Male		Female	
	Chow	HFHS	Chow	HFHS
L_PM_^s,d,i^	15.48 ± 5.42	24.80 ± 7.04	3.06 ± 2.04	2.64 ± 2.17
P_PM_^s,d^	75.76 ± 8.39	91.96 ± 11.18	30.48 ± 7.38	21.82 ± 7.94
L_PCM_^s^	8.46 ± 1.39	9.47 ± 3.97	3.62 ± 1.93	3.36 ± 2.95
P_PCM_^s^	100.32 ± 19.59	104.37 ± 19.64	38.58 ± 16.80	34.93 ± 8.24
L/P_PM_^s^	0.20 ± 0.07	0.27 ± 0.08	0.10 ± 0.07	0.11 ± 0.07
L/P_PCM_	0.45 ± 0.06	0.47 ± 0.15	0.45 ± 0.27	0.65 ± 0.68
L/P_PMGS_	0.45 ± 0.08	0.48 ± 0.07	0.39 ± 0.09	0.46 ± 0.08
L/P_PCMS_^s^	0.65 ± 0.10	0.67 ± 0.07	0.82 ± 0.14	0.84 ± 0.08
P/E_PMGS_	0.80 ± 0.07	0.79 ± 0.10	0.80 ± 0.30	0.80 ± 0.32
P/E_PCMS_^s^	0.82 ± 0.06	0.84 ± 0.08	0.90 ± 0.10	0.91 ± 0.01

## Data Availability

All raw RNA sequencing files may be obtained using GEO id: GSE228215 (https://www.ncbi.nlm.nih.gov/geo/, accessed on 7 September 2023).

## Supplementary Materials

The following supporting information can be downloaded at: https://www.mdpi.com/article/10.3390/nu15204438/s1, Figure S1: A subset of female rats in each diet group (*n* = 4) underwent a sham ovariectomy surgery (sham). The effectiveness of ovariectomy with estradiol add back (OVX + E_2_) in female rats was confirmed by measuring uterine mass relative to body weight as the uterus is sensitive to circulating estradiol and will atrophy when estradiol concentrations are low. There was no difference in sham versus OVX + E2 females for uterine mass (*p* = 0.1913). As the focus of this study was the metabolic phenotype in response to a high-fat/high-sucrose diet (HFHS), we measured glucose and insulin in the sham rats. Neither variable was different by surgery type (*p* = 0.5867 and *p* = 0.7809 respectively). Finally, we measured mitochondrial protein synthesis in sham operated rats because of the impact of estradiol on mitochondria. This variable was also unaffected by surgery (*p* = 0.5185).
