# Supplementary material for: Sex Differences in the Skeletal Muscle Response to a High Fat, High Sucrose Diet in Rats
Source: Nutrients. 2023 Oct 19;15(20):4438. doi: 10.3390/nu15204438 (PMC10610114; doi:10.3390/nu15204438)
Supplement: Supplementary file 1 [file nutrients-15-04438-s001.zip › nutrients-2629111-supplementary.pdf]

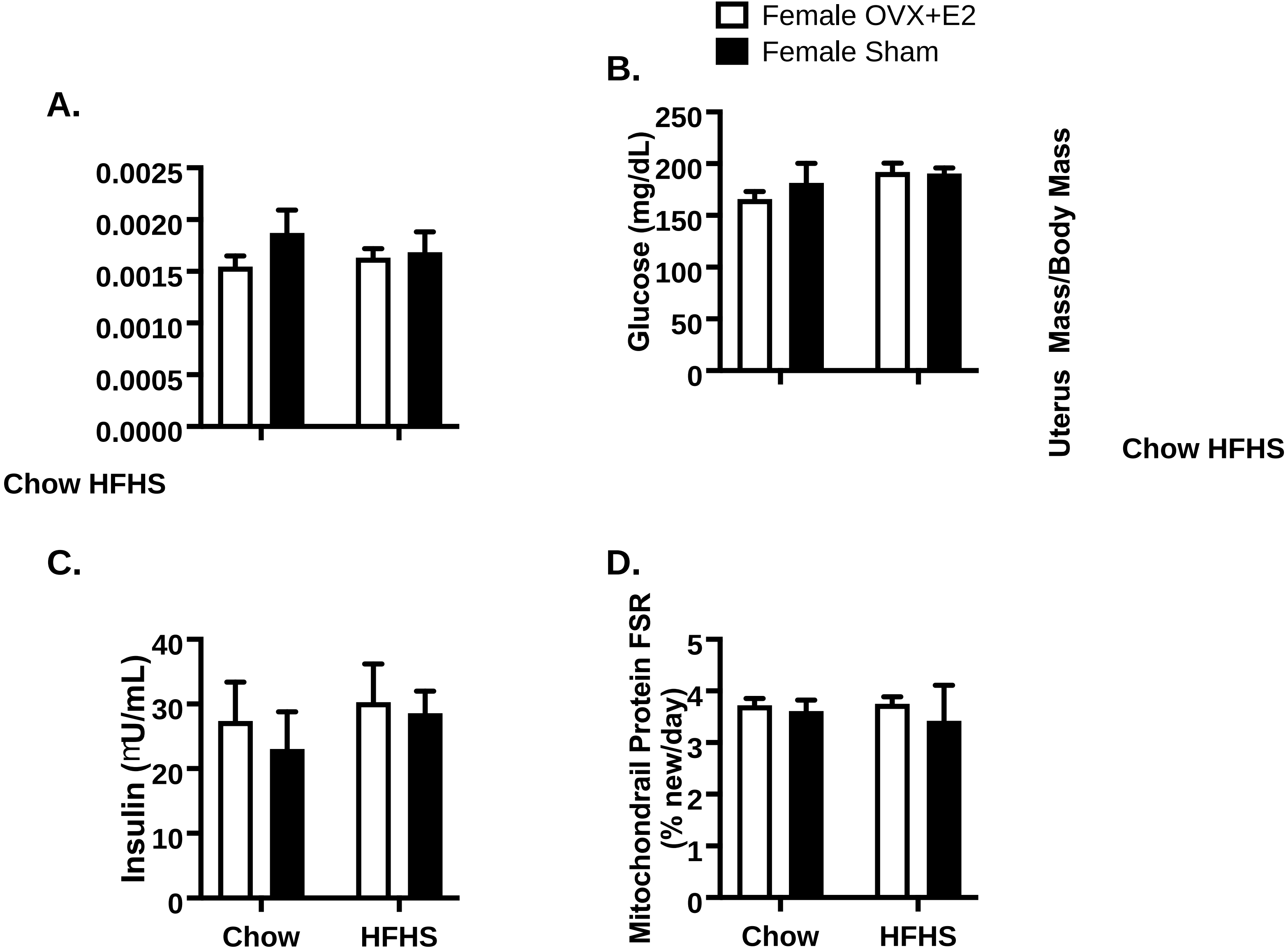

Female OVX+E2
Female Sham
A.
0.0025
0.0020
0.0015
0.0010
0.0005
0.0000
Chow HFHS
B.
Glucose (mg/dL)
250
200
150
100
50
0
Uterus Mass/Body Mass
Chow HFHS
C.
Insulin (mU/mL)
40
30
20
10
0
Chow
HFHS
D.
Mitochondrail Protein FSR
(% new/day)
5
4
3
2
1
0
Chow
HFHS

Supplementary Figure S1. A subset of female rats in each diet group (n=4) underwent a sham ovariectomy surgery (sham). The effectiveness of ovariectomy with estradiol add back (OVX+E_2) in female rats was confirmed by measuring uterine mass relative to body weight as the uterus is sensitive to circulating estradiol and will atrophy when estradiol concentrations are low. There was no difference in sham versus OVX+E_2 females for uterine mass (p=0.1913). As the focus of this study was the metabolic phenotype in response to a high-fat/high-sucrose diet (HFHS), we measured glucose and insulin in the sham rats. Neither variable was different by surgery type (p=0.5867 and p=0.7809 respectively). Finally, we measured mitochondrial protein synthesis in sham operated rats because of the impact of estradiol on mitochondria. This variable was also unaffected by surgery (p=0.5185).
